# The role of epiphytes in seagrass productivity under ocean acidification

**DOI:** 10.1038/s41598-022-10154-7

**Published:** 2022-04-15

**Authors:** Johanna Berlinghof, Friederike Peiffer, Ugo Marzocchi, Marco Munari, Grazia M. Quero, Laura Dennis, Christian Wild, Ulisse Cardini

**Affiliations:** 1 Integrative Marine Ecology Department, Stazione Zoologica Anton Dohrn – National Institute of Marine Biology, Ecology and Biotechnology, Naples, Italy; 2 Department of Marine Ecology, University of Bremen, Bremen, Germany; 3 Department of Biology, Center for Water Technology – WATEC, Aarhus University, Aarhus, Denmark; 4 Center for Electromicrobiology, Department of Biology, Aarhus University, Aarhus, Denmark; 5 Institute for Marine Biological Resources and Biotechnology, National Research Council (IRBIM-CNR), Ancona, Italy

**Keywords:** Photosynthesis, Plant symbiosis, Biogeochemistry, Biooceanography, Climate-change ecology

## Abstract

Ocean Acidification (OA), due to rising atmospheric CO_2_, can affect the seagrass holobiont by changing the plant's ecophysiology and the composition and functioning of its epiphytic community. However, our knowledge of the role of epiphytes in the productivity of the seagrass holobiont in response to environmental changes is still very limited. CO_2_ vents off Ischia Island (Italy) naturally reduce seawater pH, allowing to investigate the adaptation of the seagrass *Posidonia oceanica* L. (Delile) to OA. Here, we analyzed the percent cover of different epiphytic groups and the epiphytic biomass of *P. oceanica* leaves, collected inside (pH 6.9–7.9) and outside (pH 8.1–8.2) the CO_2_ vents. We estimated the contribution of epiphytes to net primary production (NPP) and respiration (R) of leaf sections collected from the vent and ambient pH sites in laboratory incubations. Additionally, we quantified net community production (NCP) and community respiration (CR) of seagrass communities in situ at vent and ambient pH sites using benthic chambers. Leaves at ambient pH sites had a 25% higher total epiphytic cover with encrusting red algae (32%) dominating the community, while leaves at vent pH sites were dominated by hydrozoans (21%). Leaf sections with and without epiphytes from the vent pH site produced and respired significantly more oxygen than leaf sections from the ambient pH site, showing an average increase of 47 ± 21% (mean ± SE) in NPP and 50 ± 4% in R, respectively. Epiphytes contributed little to the increase in R; however, their contribution to NPP was important (56 ± 6% of the total flux). The increase in productivity of seagrass leaves adapted to OA was only marginally reflected by the results from the in situ benthic chambers, underlining the complexity of the seagrass community response to naturally occurring OA conditions.

## Introduction

Seagrasses are among the most important marine ecosystem engineers, providing various ecosystem services and maintaining human well-being^1,2^. The habitat-forming seagrass *Posidonia oceanica*, endemic to the Mediterranean Sea, provides protection from coastal erosion, wastewater treatment and supports fisheries by providing habitats and nursery grounds for a broad range of fish and invertebrates^3,4^. *P. oceanica* meadows have high primary production rates, while decomposition rates in the seagrass sediments are rather low, creating an effective long-term carbon (C) sink^5^. Thus, *P. oceanica* meadows can be regarded as autotrophic ecosystems, releasing substantial amounts of oxygen (O_2_) while intensively sequestering carbon dioxide (CO_2_)^6^.

Coastal development and climate change have been causing a decline of 13—50% of *P. oceanica* meadows in the Mediterranean since 1960^7^. Under worst-case global warming scenarios, it is predicted that by 2050 *P. oceanica* will lose 75% of suitable habitats, and by 2100 it is at risk of functional extinction^8,9^. As a consequence of habitat degradation, and therefore, increased seagrass decomposition, the organic C stored in the sea meadows can be emitted as CO_2_ to the atmosphere^8,10^. Ocean acidification (OA, the decrease in seawater pH due to increased dissolution of atmospheric CO_2_) is an additional climate change stressor, expected to impact habitat-forming species with cascading effects on the whole marine ecosystem^11,12^. Marine calcifying organisms such as calcifying algae, corals, or mollusks are negatively affected by OA^11,13,14^. Conversely, marine macrophytes may benefit from the increased CO_2_ concentration since their photosynthetic rates are often C limited at current ocean CO_2_ levels^15^. Indeed, mesocosm studies with Zostera spp. and *Thalassia hemprichii* showed increased primary production, growth, and shoot density under increased CO_2_ availability^16–18^ and *Cymodocea nodosa* showed significantly higher seagrass productivity in naturally acidified seawater^19^. Furthermore, by removing CO_2_ from the water column through photosynthetic activity, seagrass meadows can increase pH in their surroundings, thus locally buffering OA^20,21^.

The effects of OA on *P. oceanica* remain unclear. A short-term laboratory study showed that early life stages of the plant benefit from future predicted CO_2_ concentrations and displayed bigger seed size, improved photosynthetic performance, and higher C storage in their belowground tissues^22^. However, seedlings grown under high CO_2_ concentrations were preferred by herbivorous fish, which could potentially offset the positive effects^22^. While a reduced seawater pH significantly increased the net productivity of adult plants in laboratory experiments^23^, it did not affect leaf biometrics, photosynthetic rates, and leaf growth in mesocosm experiments^24^. *P. oceanica* meadows near CO_2_ vents that have long-term adaptation to a reduced seawater pH exhibit higher shoot densities but lower leaf lengths, while their photosynthetic performance is similar at vent and ambient pH sites^25,26^.

Epiphytic algae, invertebrates, and microorganisms living in close association with the seagrass plant form a biological unit called a holobiont^27,28^. Epiphytes are key players on the seagrass phyllosphere^29^, modulating light-harvesting, gas, and nutrient exchange between the plant and the surrounding water and affecting key biogeochemical processes within the holobiont, such as C and nitrogen fixation, or transport of oxygen and dissolved organic carbon (DOC)^27,30^. Under ambient pH conditions, *P. oceanica* leaves are colonized by a large variety of epiphytes, ranging from bacteria, such as Cyanobacteria^31^ or Planctomycetes^32^, to fleshy and encrusting red, brown, and green algae^33^ and calcifying invertebrates. OA shifts the community structure from encrusting algal epiphytes to fleshy algae and non-calcifying invertebrates, such as hydrozoans and tunicates^23,26,34^. This shift in epiphyte community structure can have cascading effects on the associated communities and the functioning of the seagrass ecosystem, such as by affecting the light availability of the plant and key biogeochemical processes^27,28^.

Several studies have investigated the phenology of the epiphytic community found along pH gradients at CO_2_ vents in the field, finding reduced abundances of calcareous organisms under reduced pH^25,26,34^. However, our knowledge of the role of epiphytes in the productivity of the seagrass holobiont in response to environmental changes is still limited. The present study aims to assess the effects of OA conditions on the productivity of seagrass communities along the natural CO_2_ vents off Ischia Island and to disentangle the role of the epiphytic community vs. the plant host on seagrass productivity under OA.

## Material and methods

### Study area

The experiments were conducted in September 2019 and September 2020 at Ischia Island in the Gulf of Naples (Tyrrhenian Sea, Italy). The island is characterized by systems of submarine CO_2_ vents of volcanic origin. The gas emitted from the seafloor is composed of CO_2_ (90.1–95.3%), N_2_ (3.2–6.6%), O_2_ (0.6–0.8%), Ar (0.08–0.1%), and CH_4_ (0.2–0.8%), and it does not contain toxic sulfur compounds nor does it affect the surrounding water temperature or salinity^25,35^. One study area was located at the shallow vent system at Castello Aragonese (CA), where the vents occur at 0.5–3 m depth. Here, we selected two sites characterized by two different pH regimes (“vent pH” and “ambient pH”) at approximately 3 m water depth with similar light levels (Table 1). The vent pH site was in a venting area on the south side (40°43′50.5′′N 13°57′47.2′′E) and the ambient pH site was located on the north side of the Castello (40°43′54.8′′N 13°57′47.1′′E). Another study area for in situ incubations was located at Chiane del lume (CdL), where vents occur at 10—12 m depth (Table 1). Here, the vent pH site was located at the level of Grotta Tisichello (40°42′ 53.56′′N 13°58′ 2.37′′E) and the ambient pH site about 680 m north (40°43.248′N 13° 57.916′E).

**Table 1:** Environmental parameters (mean ± SE, *n*) measured at vent and ambient pH sites at Castello Aragonese (CA) and Chiane del Lume (CdL).

	Vent pH				Ambient pH			
	CA		CdL		CA		CdL	
Variable	Mean ± SE	*n*	Mean ± SE	*n*	Mean ± SE	*n*	Mean ± SE	*n*
T (°C)	22.95 ± 0.07	4	25.23 ± 0.02	3	22.95 ± 0.05	4	25.32 ± 0.15	3
Light (lux)	20,001 ± 271	3	4898 ± 2996	3	17,807 ± 3349	3	6213 ± 1057	3
pH	7.34 ± 0.04	2	7.92 ± 0.01	3	8.17 ± 0.02	2	8.18 ± 0.01	2
DO (mg L^-1^)	8.50 ± 0.11	4	8.87 ± 0.55	3	8.79 ± 0.22	4	8.71 ± 0.06	3
DOC (μM)	NA		143.79 ± 1.38	2	NA		139.74 ± 4.03	6
DON (μM)	NA		7.13 ± 0.06	2	NA		7.67 ± 0.83	6
NH_4_^+^	NA		0.61 ± 0.04	2	NA		0.44 ± 0.1	6
NO_3_^-^	NA		0.20 ± 0.14	2	NA		0.20 ± 0.04	6

Temperature, light, pH, and DO were continuously measured with data loggers (between 12 am and 2 pm of the respective incubation day). DOC, DON, NH_4_^+^, and NO_3_^-^ were analyzed from samples collected on the respective sampling day.

### Epiphyte NPP and R

To assess the epiphytic contribution to seagrass productivity, we collected *P. oceanica* shoots in September 2019 at the vent and ambient pH sites of Castello Aragonese and transported them directly into the laboratory. We selected leaves with homogenous coverage of epiphytes and cut off 3 cm long sections of the central part of the leaf, avoiding both young and heavily grazed and senescent parts of the plant. Epiphytes were scraped off with a scalpel from half of the leaves, taking care not to damage the plant tissue. A total of 28 *P. oceanica* leaf sections were incubated (from the vent and ambient pH sites, covered by epiphytes (+ Epi) or with epiphytes removed (− Epi), in light or dark incubation) to assess NPP and R. Leaf sections were transferred into transparent 24 ml glass vials filled with seawater from the respective pH site. The pH of the water was checked and adjusted if needed to the original site values by CO_2_ bubbling. Half of the vials were incubated in the light to assess NPP, and the others were incubated wrapped in aluminum foil to assess R. We incubated the vials on a shaker (Stuart orbital shaker SSL1; 30 rpm) under artificial light at 360 μmol m^-2^ s^-1^, upside down with the transparent bottom exposed to the light source and leaf sections standing vertically within the vials. Incubations were conducted in a temperature-controlled room at 25 °C. Oxygen concentrations were measured at the beginning and the end of the incubation (5–6 h) using a fiber-optic oxygen sensor (FireStingO2, PyroScience, Germany), making sure oxygen did not drop below 50% saturation. Temperature and pH were measured at the beginning and the end of the incubation using a pH meter (Multi 3430, WTW, Germany). PH values increased during the light incubations from 6.96 to 7.33 in the reduced pH treatment and from 8.02 to 8.25 in the ambient pH. In the dark incubations, pH values remained stable during the incubation in the reduced pH and decreased from 8.02 to 7.91 in the ambient pH. At the end of the experiment, we scraped off the epiphytes of the incubated leaf sections, and seagrass leaves and epiphytes were dried at 60 °C for 48 h and weighed separately. NPP and R were normalized to biomass (dry weight) since it reflects the different treatments (with and without epiphytes).

### NCP and CR

Natural seagrass communities were incubated in situ in September 2019 at Castello Aragonese (CA) and in September 2020 at Chiane del Lume (CdL) to assess their productivity. We estimated net community production (NCP), community respiration (CR), and nutrient fluxes during incubations with benthic chambers using the design by Olivé et al.^36^, which allows avoiding sediment disturbance, dilution, continuous stirring, or gaseous head-space while ensuring mixing through water motion^37^. The chambers consisted of an internal PVC cylinder (13 cm diameter) inserted into the plastic bag to maintain the cylindrical shape and standardize the chamber volume (10 L), a bottom cylinder inserted approximately 10–15 cm into the sediment, and a gas-tight polyethylene plastic bag with a sampling port to draw water samples. The chambers (*n* = 4) were deployed randomly within each station (CA and CdL, each with a vent and ambient pH site) by scuba divers with a minimum distance of 3 m to assure independence between the replicates. The incubations were performed during the central hours of the days, between 11.00 am and 3.00 pm. During the incubations, we measured temperature, pH, dissolved oxygen, and light intensity continuously inside the chambers, using data loggers (Onset Computer Corporation, USA). We covered the chambers with opaque polyethylene bags to exclude light and started the dark incubation to assess R. After approx. 1.5 h, we removed the covers and recorded the light incubation for another 1.5–2 h to assess NCP^36^. We collected water samples to analyze inorganic and organic nutrients with 50 ml acid-washed syringes through the sampling port immediately after the deployment of the chambers, after the dark incubation, and after the light incubation. Additionally, we took water samples from the water column inside the seagrass meadow and ca. 1 m above. The water samples were used for the analysis of dissolved inorganic nitrogen (DIN: ammonium, nitrate, and nitrite), dissolved inorganic phosphate (DIP), DOC, and dissolved organic nitrogen (DON). For DIN and DIP determination, we filtered the water through a cellulose acetate membrane filter (pore size: 0.22 μm) into 20 ml HDPE vials and stored upright at -20 °C until analysis with a Continuous Flow Analyzer (Flowsys, SYSTEA SpA., Italy). We filtered the water samples for DOC and DON determination through precombusted GF/F filters into acid-washed HDPE vials, immediately acidifying the samples with 80 µl of 18.5% HCl and storing them at 4 °C until analysis on a total organic carbon analyzer (TOC-L with TNM-L Unit, Shimadzu Corporation, Japan). We counted the total number of *P. oceanica* shoots and leaves within each incubation chamber and measured the leaf length and width in situ.

### Epiphyte biomass and community structure

We collected 20 *P. oceanica* leaves at vent pH and 20 at ambient pH sites at Castello Aragonese and directly transported them into the laboratory for community identification. We took high-resolution pictures with a stereoscope (Zeiss AxioCam 208 color) from both sides of a subset of the leaves (approx. 1 cm width and 3 cm length). We analyzed the community structure by identifying major groups and estimated percent cover using the software CPCe 4.1, counting 25 random points per frame (20 leaves per site × 2 sides of the leaf = 80 frames in total). Subsequently, we carefully scraped off the epiphytes with a scalpel, dried the leaves and epiphytes at 60 °C for 48 h, and weighed them separately to estimate the *P. oceanica* leaf and epiphyte biomass.

### Data analysis

NPP and R rates in the laboratory incubations were calculated as:

$$ NPP\, or\, R,\ \left( {\mu M\, O_{2}\, g^{ - 1} h^{ - 1} } \right) = \frac{{\left( {\left[ {O_{2} } \right]_{final} - \left[ {O_{2} } \right]_{initial} } \right)*V}}{DW*t} $$

where [O_2_] is the oxygen concentration (µmol L^-1^) in the light (NPP) and the dark (R) incubations, *V* is the volume of the vials (24 mL), *DW* is the dry weight of the seagrass leaf biomass (g), and *t* is the incubation time (h). We tested the effects of pH (vent pH vs. ambient pH), treatment (-Epi vs. + Epi), and their interaction on the productivity in a two-way ANOVA (Type II) and used estimated marginal means (EMMs) for posthoc pairwise comparison of the fitted means. We tested for normality and homogeneity of variances before each analysis using Shapiro–Wilk's and Levene's tests. ANOVA Type II was performed despite the unbalanced design, as the test is considered robust to moderate departures from unequal sample sizes when the homogeneity of variances is met^38^.

In situ NCP and CR were calculated as:

$$ NCP\, or\, CR\, \left( {mM\, O_{2}\, m^{ - 2} h^{ - 1} } \right) = \frac{\Delta DO*V}{A} $$

where *∆DO* is the slope obtained from the linear regression of the oxygen concentrations (mmol L^-1^ h^-1^) during the light (NCP) and dark (CR) incubations, *V* is the volume of the benthic chamber (10 L), and *A* is the chamber area (0.013 m^2^).

Gross primary production (GPP) was calculated as:

$$ GPP\, \left( {mM\, O_{2}\, m^{ - 2} h^{ - 1} } \right) = NPP + R $$

The daylight NCP and night CR budgets were calculated from the NCP and CR rates during 24 h, considering an 11:13 light/darkness photoperiod. NCP daily budgets were calculated as the sum of daylight NCP and night CR budgets according to Olivé et al. (2016). We tested the effects of the pH (vent pH vs. ambient pH) on the community productivity, on the total epiphyte cover and the percent cover of the individual epiphytic groups using one-way ANOVAs (Type II). We tested for normality and homogeneity of variances before each analysis using Shapiro–Wilk's and Levene's tests and removed outliers and used generalized linear models (GLM) with Poisson or Quasi Poisson distribution when normality and homogeneity were not met. All statistical analyses were performed with RStudio (version 3.5.3) using the packages *car*, *ggplot2*, and *emmeans*^39^.

### Research involving plants

The authors declare that the study have been carried out in accordance with relevant guidelines and regulations.

## Results

### Epiphyte biomass and community structure

**Figure 1:**
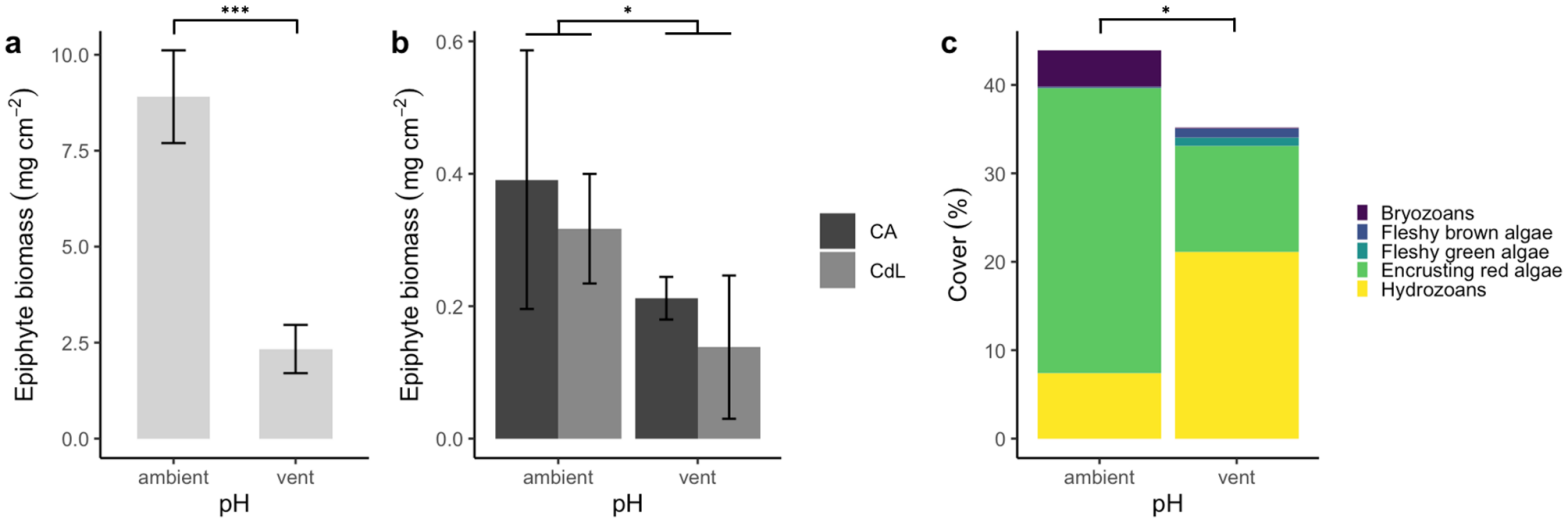
Epiphyte biomass and community structure. (**a**) Epiphyte biomass per leaf section from vent and ambient pH sites in the laboratory incubations, (**b**) epiphyte biomass (whole leaf) per leaf area in the benthic chambers at vent and ambient pH sites at Castello Aragonese (CA) and Chiane del Lume (CdL), (**c**) percent cover of the epiphytic groups at Chiane del Lume (only species groups with > 1% cover are shown). Error bars indicate 95% confidence intervals. Stars show significant differences; number of stars show significance level (*p < 0.05, **p < 0.01, ***p < 0.001).

The epiphytic biomass of the leaf sections in the laboratory incubations (Fig. 1a) was 2.8-fold higher at ambient pH sites compared to vent pH sites (F_1,13_ = 96.52, *p* < 0.001, R^2^ = 0.87). The epiphytic biomass of the leaves collected from the benthic chambers after the in situ incubations (Fig. 1b) was on average 0.7-fold higher at ambient pH sites compared to vent pH sites (F_1,11_ = 9.48, *p* = 0.011, R^2^ = 0.37). The total epiphyte cover and the community composition differed between vent and ambient pH sites (Fig. 1c). Leaves from ambient pH sites showed a 25% higher total epiphyte cover (F_1,78_ = 4.20, *p* = 0.043, R^2^ = 0.04) and were mainly covered with encrusting red algae (32%) followed by hydrozoans (7%). Leaves from vent pH sites were primarily covered with hydrozoans (21%), followed by encrusting red algae (12%). The coverage of calcifying groups such as bryozoans and encrusting red algae was higher at ambient pH sites, namely 41 × higher for bryozoans (GLM, Chisq = 18.21, df = 1, *p* < 0.001) and 2.7 × higher for encrusting red algae (GLM, Chisq = 32.27, df = 1, *p* < 0.001). Non-calcifying groups, such as fleshy green algae and hydrozoans, showed higher coverage at vent pH sites. Fleshy green algae (GLM, Chisq = 4.47, df = 1, *p* = 0.034) were only present at vent pH sites and the coverage of hydrozoans was 2.9 × increased at vent pH sites (GLM, Chisq = 21.991, df = 1, *p* < 0.001).

### Contribution of epiphytes to seagrass productivity in laboratory incubations

**Figure 2:**
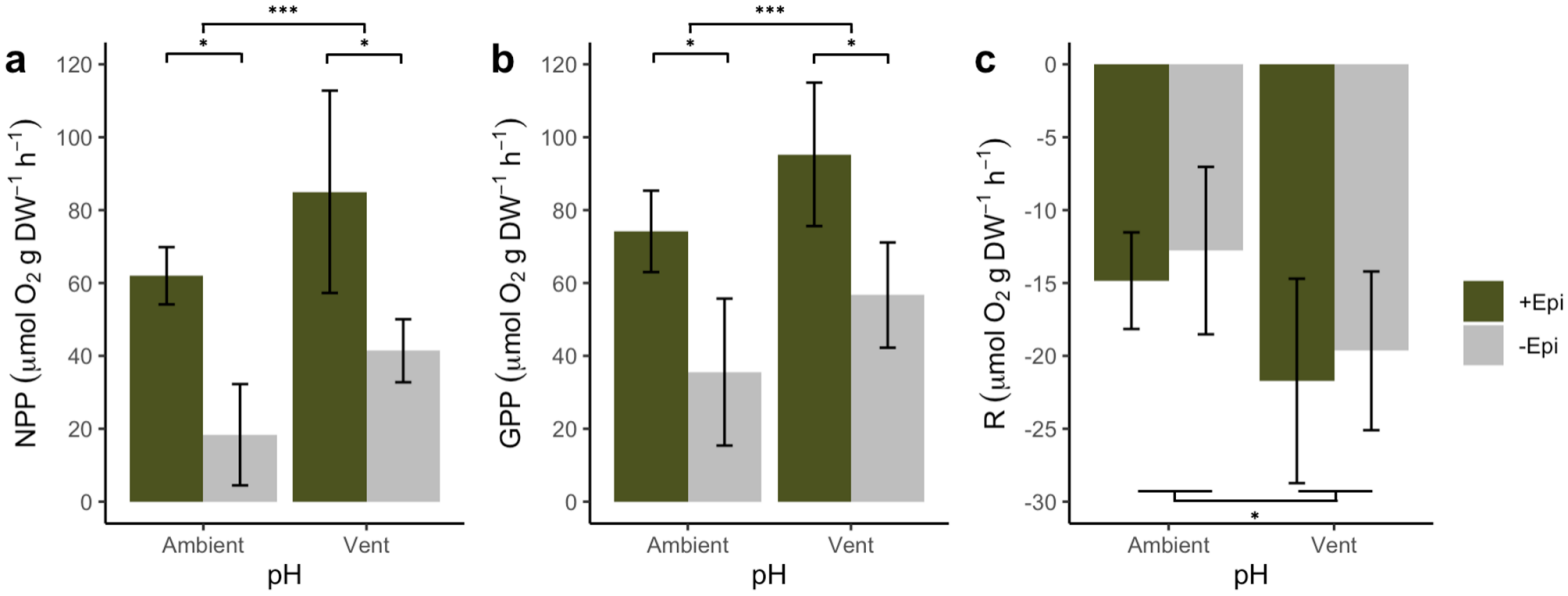
Ex situ net primary production (**a**), gross primary production (**b**), and respiration (**c**) of leaves with epiphytes (+ Epi, green) and without epiphytes (− Epi, grey), from vent and ambient pH sites, normalized by seagrass leaf biomass (dry weight). Negative values represent oxygen consumption, while positive values show oxygen production. Error bars indicate 95% confidence intervals. Stars show significant differences; number of stars show significance level (*p < 0.05, **p < 0.01, ***p < 0.001).

We found that NPP (Fig. 2a) significantly increased in the presence of epiphytes (F_1,10_ = 51.92, *p* < 0.001, R^2^ = 0.82), which contributed on average 56 ± 6% (mean ± SE) to NPP regardless of pH. At the same time, NPP increased on average by 47 ± 21% in leaves from the vent pH site (F_1,10_ = 8.81, *p* = 0.014, R^2^ = 0.82) compared to the ambient pH site, regardless of presence/absence of epiphytes. GPP (Fig. 2b) followed a similar pattern and increased in the presence of epiphytes (F_1,10_ = 23.60, *p* < 0.001, R^2^ = 0.69) and in leaves from the vent pH site (F_1,10_ = 7.07, *p* = 0.024, R^2^ = 0.69). Respiration (Fig. 2c) was not affected by the presence/absence of epiphytes but increased on average by 50 ± 4% in leaves from the vent pH site (F_1,11_ = 5.80, *p* = 0.035, R^2^ = 0.25) compared to the ambient pH site.

### In situ net community production and respiration

To assess the productivity of natural seagrass communities under OA, we measured in situ oxygen production in light vs. dark incubations at vent vs. ambient pH sites. We observed no significant differences between daylight, night, or daily budgets at the vent and ambient pH sites at Castello Aragonese or Chiane del Lume (Table 2).

**Table 2:** Daily metabolic budgets (mean ± SE) of P. oceanica at vent and ambient pH sites at Castello Aragonese (CA) and Chiane del Lume (CdL).

	Location	*n*	Daylight budget (mmol O_2_ m^-2^)	Night budget (mmol O_2_ m^-2^)	Daily budget (mmol O_2_ m^-2^ day^-1^)
Vent pH	CA	4	243.90 ± 60.07	− 165.34 ± 18.99	70.60 ± 55.45
	CdL	3	233.89 ± 35.55	− 200.07 ± 56.81	33.81 ± 24.10
Ambient pH	CA	4	207.52 ± 34.55	− 176.88 ± 22.99	30.63 ± 20.10
	CdL	3	211.68 ± 19.73	− 201.02 ± 41.23	10.66 ± 23.25
Olivé et al.^36^	Revellata Bay (Calvi, France)	3	143.34 ± 12.53	− 81.68 ± 6.51	61.67 ± 14.12

Daylight budget was calculated from net community production (NCP) and night budget from community respiration (CR), assuming an 11:13 light: dark cycle. Negative values mean a net consumption of O_2_. The daily budget was calculated as the sum of daylight and night budget. The budgets calculated by Olivé et al. (2016) after 1.5-2 h incubation time were added for comparison.

DOC, DON, NH_4_^+^, NO_2_^-^, and NO_3_^-^ fluxes did not change during dark and light incubations and were not affected by seawater pH (Suppl. Table 1). The PO_4_^-^ flux slightly decreased during the light incubations at the vent and ambient pH sites by 0.019 ± 0.0005 (mean ± SE) μM h^-1^.

The morphology of the seagrass meadows differed significantly between the two sites (Suppl. Table 2). The seagrass at the vent sites displayed a higher shoot density but shorter average leaf length and width, causing the leaf area index not to differ significantly between the vent and ambient pH sites.

## Discussion

### Epiphytic communities differ between seagrass leaves from vent and ambient pH sites

The epiphytic communities in the studied vent area at Chiane del Lume showed significant differences among pH conditions. The overall epiphyte cover was 25% higher under ambient pH conditions. Encrusting red algae showed a reduced coverage from 32% under ambient pH conditions to 12% under vent pH conditions. The coverage of the non-calcifying hydrozoans increased from 7 to 21% under OA conditions. This shift from coralline to non-calcifying organisms was also found by Mecca et al. (2020) in the vent system of Castello Aragonese. Several studies showed that encrusting red algae (Corallinales) are especially vulnerable to acidification due to the sensitivity of their carbonate skeleton^25,34,40^. On the other side, hydrozoans show a higher tolerance to reduced seawater pH and can, therefore, outcompete more pH-sensitive species^41^. In contrast to Mecca et al. (2020), we found that also bryozoans were negatively affected by low pH conditions. Bryozoans are calcifying organisms as well, but due to organic tissue protecting their skeleton and different mineralogical composition, they are less sensitive to OA as coralline algae. However, Rodolfo-Metalpa et al.^42^ found reduced calcification rates under very low pH conditions (pH 7.43) and high mortality rates when low pH was combined with high seawater temperatures (25–28 °C).

The epiphytic biomass was significantly higher at leaf sections from ambient pH sites. This is attributable to the higher epiphytic coverage at ambient pH sites and the difference in epiphytic calcium carbonate mass^34^. The differences in epiphytic biomass between the vent and ambient pH sites of seagrass leaves collected from within the benthic chambers were not as pronounced as of leaf sections in the laboratory incubations (0.7-fold increase instead of 2.8-fold increase at vent pH sites) and displayed lower values. This results from high variability in epiphytic growth on *P. oceanica* leaves in situ, including young (non-epiphytized) and senescent portions that were excluded from the laboratory incubations.

### Epiphytes contribute to leaf NPP under vent and ambient pH conditions

In our laboratory incubations, epiphytes accounted for 50% of *P. oceanica* leaf NPP under vent pH and 62% under ambient pH conditions. Several studies have found that epiphytes contribute up to 60% to photosynthesis and primary production for different seagrass species, such as *Halodule wrightii, Syringodium filiforme, Thalassia testudinum*^43^, and *Zostera marina*^44^. The epiphytic community of *P. oceanica* can be highly diverse, with 430 epiphyte species recorded on its leaves^45^. These are accompanied by a diverse prokaryotic community within the leaf biofilm^32^. Among this community, many members are phototrophs, such as the abundant Corallinales, Ochrophyta, Chlorophyta, diatoms, and cyanobacteria^45^. In leaves from the ambient pH site, the bulk of the measured epiphytic NPP on the leaves was likely attributable to Corallinales, which covered large portions of the leaf surface and are found to be the most abundant epiphytic group (ca. 30% cover) at ambient pH around the Castello Aragonese in Ischia^26^. Conversely, epiphytic organisms other than Corallinales are likely responsible for the contribution to NPP in leaves from the vent pH site. Within the diverse epiphytic consortium, heterotrophic bacteria can also indirectly contribute to primary production, helping to overcome the shortcoming of limiting nitrogen and phosphorous^46^. However, with our experimental approach, it was not possible to determine micro-epiphytes that occur within the biofilm of the leaf surface, such as cyanobacteria, dinoflagellates, foraminifers, or planctomycetes^32,45^. Albeit, these epiphytic communities can also turn into a threat to the plant if coastal eutrophication and global warming result in their overgrowth on the seagrass phyllosphere^47,48^. In these cases, leaf epiphytes can lead to a strong O_2_ build-up, increased oxidative stress, reduced light conditions in the leaf micro-environment in the light, or reduced internal plant aeration and production of phytotoxic nitric oxide in the dark^48,49^. Moreover, thick biofilms can thermally stress the underlying plan leaf tissue when the seagrass is already close to its upper thermal limits^48^.

While epiphytes clearly drove NPP in our laboratory incubations, R was not affected by the presence/absence of epiphytes. This is in agreement with the results of Costa et al. (2015), who observed no effects of epiphytes on R of *P. oceanica* shoots. By contrast, Brodersen et al. (2020) found lower R rates in leaves of *Zostera marina* with epiphytes as a consequence of the reduced diffusive O_2_ uptake of epiphyte-covered seagrass leaves.

### NPP and R of seagrass leaf sections increases under OA conditions

The relationship between decreasing pH and increasing production of *P. oceanica* has been investigated over a wide range of pH from 7.9 to 5.5^23,25,50^, using a variety of methods. Other seagrass species, such as Zostera spp*.*^17,51^, *Thalassia hemprichii*^18^, and *Cymodocea nodosa*^19^ also showed stimulation in productivity under lower pH conditions. NPP, GPP, and R were significantly higher in leaves from vent pH sites in our laboratory experiments. On average, NPP increased by 47 ± 21% (mean ± SE) and R by 50 ± 4%, suggesting that the *P. oceanica* holobiont is indeed C-limited at current seawater inorganic C concentrations. However, increased seagrass productivity is not necessarily expected to translate into net growth of the meadow. Accordingly, an increased vulnerability of *P. oceanica* leaves to grazing by herbivores^26^ is attributed to the more labile organic composition of the seagrass holobiont^52^ and, as our data indicate, by the absence of calcareous epiphytes at vent pH sites. Additionally, so far it is not entirely clear whether it is the plant or its epiphytes that are mainly benefiting from the increased CO_2_ concentrations. Since epiphytic fleshy algae respond positively to increased CO_2_ availability^15^, they could compete with their plant host for similar resources under OA conditions. Hansen et al. showed that epiphytes of the seagrass *Zostera marina* can have a competitive advantage under elevated CO_2_ at seawater temperatures up to 22 °C. Additionally, epiphytic biofilms reduced the photosynthetic efficiency of the seagrass especially under higher temperatures (27 °C)^53^. Competition between seagrasses and filamentous algal epiphytes has been also shown under high CO_2_ and high light^54^ as well as in polluted conditions^55^. In our laboratory incubations, epiphytic contribution to NPP was 62% in leaves from the ambient pH site, while 50% in those from the vent pH site. Furthermore, NPP of leaves from the vent pH site was higher than NPP of leaves from the ambient pH site by 26% with epiphytes present and by 68% with epiphytes removed. While the plant directly benefits from increased CO_2_ concentrations and reduced shading by calcareous epiphytes, the lower epiphytic contribution to NPP in the CO_2_ vents is likely a combined result of changes in biomass, community composition as well as species-specific rates.

### Productivity of the seagrass community is only marginally affected by OA

*P. oceanica* meadows at the vent pH sites showed higher shoot density but shorter leaf length and width than at ambient pH sites (Suppl. Table 2). Increased shoot density and shorter leaf length under vent pH conditions have been reported for *P. oceanica* and other seagrass species^25,34,51^. These changes in seagrass morphology under OA have been associated with an increased grazing pressure by herbivores, such as the fish *Sarpa salpa*, sea urchins, or other invertebrates^22,56^. As a reaction to high grazing activity, *P. oceanica* invests energy-rich compounds produced by photosynthesis into shoot recruitment rather than belowground C storage^52^. Fluxes of organic (DOC, DON) and inorganic (NH_4_^+^, NO_2_^−^, NO_3_^−^) nutrients did not differ between vent and ambient pH sites in the dark and the light incubations (Suppl. Table 1) but showed high variability among the benthic chambers. PO_4_^−^ consumption was higher during light incubations than dark incubations at both vent and ambient pH sites. Phosphate is essential for effective photosynthesis and therefore actively taken up by seagrasses^57^. Our estimates for *P. oceanica* metabolic daylight, night, and daily budgets are in the same order of magnitude as those reported by Olivé et al.^36^ using similar chambers and incubation times. While our daily budgets agree well with their results, we found higher daylight and night budgets. This can be an effect of different light intensities during the incubations and different plant biomasses within the incubation chambers. Our incubations were carried out in September, while Olivé and colleagues carried out their incubations in October in Calvi (France), at a higher latitude than our station in Ischia (Italy). Despite differences in morphology, and differently from what we have reported for our laboratory experiments, there was no statistically significant increase in productivity of in situ seagrass communities at the vent pH sites. However, we saw a pattern of higher autotrophy at the vent pH sites of CA and CdL compared to the respective ambient pH sites, which resulted in more than two-fold average daily budgets under OA conditions. When normalizing the in-situ productivity to biomass (Suppl. Fig. 1), we saw a pattern of higher productivity and respiration at the vent site of CA. In contrast, productivity did not differ between the ambient and vent sites of CdL. The different patterns of in-situ productivity between the two locations are probably a result of their differences in depth and hence light intensity as well as the different bubbling intensity of the CO_2_ vents and, therefore, pH ranges. The location of CdL is deeper (10–12 m) than CA (3 m), resulting in a threefold lower light intensity (see Table 1). At CdL, the pH range between the ambient and vent site is not as high as for CA (7.92–8.18 and 7.34–8.17, respectively). Eventually, high variability in benthic metabolism prevented discerning significant differences. When logistically feasible, follow-up studies should thus consider an increased replication when measuring benthic metabolism *in-situ*.

Seagrasses are not only colonized by epiphytes living on the leaf surface but also at the roots and rhizomes of the plant^27,45^. Additionally, various phototrophic and heterotrophic organisms inhabit the *P. oceanica* belowground habitat^58^. These organisms were unaccounted for in our laboratory experiments while they were included in the in situ benthic incubation chambers. A recent study on rocky benthic communities from the same CO_2_ vents in Ischia found functional vulnerability (i.e., decrease in functional diversity following the loss of species) to OA to be more pronounced than the corresponding decrease in taxonomic diversity, identifying heterotrophic feeding strategies among the functional entities that are most vulnerable to OA^59^. If similar scenarios apply to the *P. oceanica* communities, this may explain our results, suggesting increased autotrophy at the vents. This, however, may not translate into more C sequestration, as the more labile organic composition of the seagrass holobiont^52^ and the absence of calcareous epiphytes at vent pH sites leads to increased grazing and C remineralization. Additional experiments with more replication throughout the year would provide helpful insights about seasonal patterns that might occur.

## Conclusions

In summary, the present study demonstrates that natural CO_2_ enrichment clearly affects the epiphyte community structure and the productivity of both seagrass leaves and their epiphytic community. Epiphytes contributed significantly to NPP under vent and ambient pH conditions but not to seagrass respiration. However, this was only marginally translated to changes in NCP or CR at the community level in situ. Our results show the high complexity of host-epiphyte interactions and their response to environmental changes such as OA. A comparison with other studies shows that this response is highly dependent upon spatial and temporal scales, the species themselves, and environmental characteristics of the site. However, it is clear that studies that seek to understand seagrass biology and ecology cannot disregard the role of its associated epiphytes.

## Supplementary Information

Supplementary Information.

## Data Availability

The datasets generated during and analyzed during the current study are available from the corresponding author on reasonable request.

## References

[1] Cullen-Unsworth LC (2014). Seagrass meadows globally as a coupled social-ecological system: Implications for human wellbeing. Mar. Pollut. Bull..

[2] Ondiviela B (2014). The role of seagrasses in coastal protection in a changing climate. Coast. Eng..

[3] Campagne CS, Salles J-M, Boissery P, Deter J (2015). The seagrass Posidonia oceanica: ecosystem services identification and economic evaluation of goods and benefits. Mar. Pollut. Bull..

[4] Boudouresque CF, Mayot N, Pergent G (2006). The outstanding traits of the functioning of the Posidonia oceanica seagrass ecosystem. Biol. Mar. Medit..

[5] Duarte CM, Kennedy H, Marbà N, Hendriks I (2013). Assessing the capacity of seagrass meadows for carbon burial: Current limitations and future strategies. Ocean Coast. Manag..

[6] Barrón C, Duarte CM, Frankignoulle M, Borges AV (2006). Organic carbon metabolism and carbonate dynamics in a mediterranean seagrass (Posidonia oceanica) Meadow. Estuar. Coasts.

[7] Marbà N, Díaz-Almela E, Duarte CM (2014). Mediterranean seagrass (Posidonia oceanica) loss between 1842 and 2009. Biol. Conserv..

[8] Chefaoui RM, Duarte CM, Serrão EA (2018). Dramatic loss of seagrass habitat under projected climate change in the Mediterranean Sea. Glob. Chang. Biol..

[9] Marbà N, Duarte CM (2010). Mediterranean warming triggers seagrass (Posidonia oceanica) shoot mortality. Glob. Chang. Biol..

[10] Lovelock CE (2017). Assessing the risk of carbon dioxide emissions from blue carbon ecosystems. Front Ecol Env..

[11] Kroeker KJ (2013). Impacts of ocean acidification on marine organisms: quantifying sensitivities and interaction with warming. Glob. Chang. Biol..

[12] Zunino S, Libralato S, Canu DM, Prato G, Solidoro C (2021). Impact of ocean acidification on ecosystem functioning and services in habitat-forming species and marine ecosystems. Ecosystems.

[13] Riebesell U (2000). Reduced calcification of marine plankton in response to increased atmospheric CO2. Nature.

[14] Doney SC, Fabry VJ, Feely RA, Kleypas JA (2009). Ocean acidification: the other CO_2_ problem. Annu. Rev. Mar. Sci..

[15] Koch M, Bowes G, Ross C, Zhang X-H (2013). Climate change and ocean acidification effects on seagrasses and marine macroalgae. Glob. Chang. Biol..

[16] Zimmerman RC (2017). Experimental impacts of climate warming and ocean carbonation on eelgrass Zostera marina. Mar. Ecol. Prog. Ser..

[17] Egea LG, Jimé Nez-Ramos R, Herná Ndez I, Bouma TJ, Brun FG (2018). Effects of ocean acidification and hydrodynamic conditions on carbon metabolism and dissolved organic carbon (DOC) fluxes in seagrass populations. PLoS ONE.

[18] Jiang ZJ, Huang X-P, Zhang J-P (2010). Effects of CO 2 enrichment on photosynthesis, growth, and biochemical composition of seagrass thalassia hemprichii (ehrenb.) aschers. J. Integr. Plant Biol..

[19] Apostolaki ET, Vizzini S, Hendriks IE, Olsen YS (2014). Seagrass ecosystem response to long-term high CO_2_ in a Mediterranean volcanic vent. Mar. Environ. Res..

[20] Hendriks IE (2014). Photosynthetic activity buffers ocean acidification in seagrass meadows. Biogeosciences.

[21] Bergstrom E, Silva J, Martins C, Horta P (2019). Seagrass can mitigate negative ocean acidification effects on calcifying algae. Sci. Rep..

[22] Hernán G (2016). Seagrass (Posidonia oceanica) seedlings in a high-CO 2 world: from physiology to herbivory. Sci. Rep..

[23] Cox TE (2015). Effects of ocean acidification on Posidonia oceanica epiphytic community and shoot productivity. J. Ecol..

[24] Cox TE (2016). Effects of in situ CO2 enrichment on structural characteristics, photosynthesis, and growth of the Mediterranean seagrass Posidonia oceanica. Biogeosciences.

[25] Hall-Spencer JM (2008). Volcanic carbon dioxide vents show ecosystem effects of ocean acidification. Nature.

[26] Mecca S, Casoli E, Ardizzone G, Gambi MC (2020). Effects of ocean acidification on phenology and epiphytes of the seagrass Posidonia oceanica at two CO2 vent systems of Ischia (Italy). Mediterr. Mar. Sci..

[27] Ugarelli K, Chakrabarti S, Laas P, Stingl U (2017). The seagrass holobiont and its microbiome. Microorganisms.

[28] Tarquinio F, Hyndes GA, Laverock B, Koenders A, Säwström C (2019). The seagrass holobiont: understanding seagrass-bacteria interactions and their role in seagrass ecosystem functioning. FEMS Microbiol. Lett..

[29] Brodersen KE, Kühl M (2022). Effects of Epiphytes on the Seagrass Phyllosphere. Front. Mar. Sci..

[30] Seymour, J. R., Laverock, B., Nielsen, D. A., M., T.-T. S. & Macreadie, P. I. The Microbiology of Seagrasses. in *Seagrasses of Australia* 343–392 (Springer International Publishing, 2018). 10.1007/978-3-319-71354-0

[31] Ruocco N (2018). First evidence of Halomicronema metazoicum (Cyanobacteria) free-living on Posidonia oceanica leaves. PLoS ONE.

[32] Kohn T (2020). The microbiome of posidonia oceanica seagrass leaves can be dominated by planctomycetes. Front. Microbiol.

[33] Casola E, Scardi M, Mazzella L, Fresi E (1987). Structure of the epiphytic community of posidonia oceanica leaves in a shallow meadow. Mar. Ecol..

[34] Martin S (2008). Effects of naturally acidified seawater on seagrass calcareous epibionts. Biol. Lett.

[35] Foo SA, Byrne M, Ricevuto E, Gambi MC (2018). The carbon dioxide vents of Ischia, Italy, a natural system to assess impacts of ocean acidification on marine ecosystems: an overview of research and comparisons with other vent systems. Oceanogr. Mar. Biol..

[36] Olivé I, Silva J, Costa MM, Santos R (2016). Estimating seagrass community metabolism using benthic chambers: the effect of incubation time. Estuaries Coasts.

[37] Barrón C, Duarte CM (2009). Dissolved organic matter release in a Posidonia oceanica meadow. Mar. Ecol. Prog. Ser..

[38] Langsrud Ø (2003). ANOVA for unbalanced data: Use Type II instead of Type III sums of squares. Stat. Comput..

[39] RStudio Team. RStudio. *R: A Language and Environment for Statistical Computing. R Foundation for Statistical Computing, Vienna, Austria. URL *https://www.R-project.org/*.* (2021).

[40] Donnarumma L, Lombardi C, Cocito S, Gambi MC (2014). Settlement pattern of Posidonia oceanica epibionts along a gradient of ocean acidification: an approach with mimics. Mediterr. Mar. Sci..

[41] Gravili C, Cozzoli F, Gambi MC (2021). Epiphytic hydroids on Posidonia oceanica seagrass meadows are winner organisms under future ocean acidification conditions: evidence from a CO2 vent system (Ischia Island, Italy). Eur. Zool. J..

[42] Rodolfo-Metalpa R, Lombardi C, Cocito S, Hall-Spencer JM, Gambi MC (2010). Effects of ocean acidification and high temperatures on the bryozoan Myriapora truncata at natural CO2 vents. Mar. Ecol..

[43] Wear DJ, Sullivan MJ, Moore AD, Millie DF (1999). Effects of water-column enrichment on the production dynamics of three seagrass species and their epiphytic algae. Mar. Ecol. Prog. Ser..

[44] Hasegawa N, Hori M, Mukai H (2007). Seasonal shifts in seagrass bed primary producers in a cold-temperate estuary: Dynamics of eelgrass Zostera marina and associated epiphytic algae. Aquat. Bot..

[45] Piazzi L, Balata D, Ceccherelli G (2016). Epiphyte assemblages of the Mediterranean seagrass Posidonia oceanica: an overview. Mar. Ecol..

[46] Celdrán D, Espinosa E, Sánchez-Amat A, Marín A (2012). Effects of epibiotic bacteria on leaf growth and epiphytes of the seagrass Posidonia oceanica. Mar. Ecol. Prog. Ser..

[47] Brodersen KE, Koren K, Revsbech NP, Kühl M (2020). Strong leaf surface basification and CO2 limitation of seagrass induced by epiphytic biofilm microenvironments. Plant Cell Environ..

[48] Noisette F, Depetris A, Kühl M, Brodersen KE (2020). Flow and epiphyte growth effects on the thermal, optical and chemical microenvironment in the leaf phyllosphere of seagrass (Zostera marina). J. R. Soc. Interface.

[49] Costa MM (2015). Epiphytes modulate posidonia oceanica photosynthetic production, energetic balance, antioxidant mechanisms, and oxidative damage. Front. Mar. Sci..

[50] Guilini K (2017). Response of Posidonia oceanica seagrass and its epibiont communities to ocean acidification. PLoS ONE.

[51] Palacios SL, Zimmerman RC (2007). Response of eelgrass Zostera marina to CO2 enrichment: possible impacts of climate change and potential for remediation of coastal habitats. Mar. Ecol. Prog. Ser..

[52] Scartazza A (2017). Carbon and nitrogen allocation strategy in Posidonia oceanica is altered by seawater acidification. Sci. Total Environ..

[53] Hansen, A. B., Pedersen, A. S., Kühl, M. & Brodersen, K. E. Temperature Effects on Leaf and Epiphyte Photosynthesis, Bicarbonate Use and Diel O 2 Budgets of the Seagrass Zostera marina L. *Front. Mar. Sci.* **9**, (2022).

[54] Burnell OW, Russell BD, Irving AD, Connell SD (2014). Seagrass response to CO2 contingent on epiphytic algae: indirect effects can overwhelm direct effects. Oecologia.

[55] Mabrouk L, Hamza A, Brahim B, Bradai M-N (2013). Variability in the structure of epiphyte assemblages on leaves and rhizomes of Posidonia oceanica in relation to human disturbances in a seagrass meadow off Tunisia. Aquat. Bot..

[56] Garrard SL (2014). Indirect effects may buffer negative responses of seagrass invertebrate communities to ocean acidification. J. Exp. Mar. Bio. Ecol..

[57] Touchette BW, Burkholder JAM (2000). Review of nitrogen and phosphorus metabolism in seagrasses. J. Exp. Mar. Bio. Ecol..

[58] Borg JA, Rowden AA, Attrill MJ, Schembri PJ, Jones MB (2006). Wanted dead or alive: high diversity of macroinvertebrates associated with living and ‘dead’ Posidonia oceanica matte. Mar. Biol..

[59] Teixidó N (2018). Functional biodiversity loss along natural CO 2 gradients. Nat. Commun..

