# Supplementary material for: The role of epiphytes in seagrass productivity under ocean acidification
Source: Sci Rep. 2022 Apr 15;12:6249. doi: 10.1038/s41598-022-10154-7 (PMC9012757; doi:10.1038/s41598-022-10154-7)
Supplement: Supplementary file 1 — Supplementary Information. [file 41598_2022_10154_MOESM1_ESM.pdf]

Supplementary material

Title

The Role of Epiphytes in Seagrass Productivity under Ocean Acidification

Authors

Johanna Berlinghof[1,2*], Friederike Peiffer[1,2], Ugo Marzocchi[1,3,4], Marco Munari[1], Grazia M. Quero[5], Laura Dennis[1], Christian Wild[2], Ulisse Cardini[1*]

[1]Integrative Marine Ecology Department, Stazione Zoologica Anton Dohrn – National Institute of Marine Biology, Ecology and Biotechnology, Italy

[2]Department of Marine Ecology, University of Bremen, Germany

[3]Center for Water Technology – WATEC, Department of Biology, Aarhus University, Denmark

[4]Center for Electromicrobiology, Department of Biology, Aarhus University, Denmark

[5]Institute for Marine Biological Resources and Biotechnology, National Research Council (IRBIM-CNR), Ancona, Italy

Supplementary Table 1. Organic and inorganic nutrient fluxes (mean ± SE) during dark and light incubations at acidified and control pH sites of Chiane del Lume. ANOVA results testing the differences between sites and dark/light incubation are given.

Variable (μM h^{-1})	Vent pH (mean ± SE)		Ambient pH (mean ± SE)		ANOVA	Sum of squares	*F*-value	*p*-value
	Dark (*n*=4)	Light (*n*=3)	Dark (*n*=7)	Light (*n*=7)				
DOC	21.85 ± 19.28	19.30 ± 13.39	23.05 ± 14.63	20.50 ± 15.01	Site (df=1)	5.9	0.01	ns
					Dark/light (df=1)	27.3	0.03	ns
DON	-1.14 ± 1.46	0.34 ± 1.14	-3.74 ± 2.05	-2.26 ± 1.83	Site (df=1)	31.36	1.57	ns
					Dark/light (df=1)	11.44	0.52	ns
NH_4^+	0.10 ± 0.46	-0.31 ± 0.17	-0.12 ± 0.24	-0.54 ± 0.15	Site (df=1)	0.23	0.69	ns
					Dark/light (df=1)	0.91	2.73	ns
PO_4^-	0.006 ± 0.004	-0.019 ± 0.017	0.007 ± 0.013	-0.018 ± 0.007	Site (df=1)	0.000005	0.01	ns
					Dark/light (df=1)	0.003	4.56	0.047
NO_2^-	0.023 ± 0.028	0.007 ± 0.021	0.019 ± 0.027	0.003 ± 0.012	Site (df=1)	0.00007	0.02	ns
					Dark/light (df=1)	0.001	0.45	ns
NO_3^-	0.17 ± 0.08	0.13 ± 0.08	-0.02 ± 0.19	-0.06 ± 0.07	Site (df=1)	0.16	1.34	ns
					Dark/light (df=1)	0.01	0.07	ns

Supplementary Table 2. Morphological traits (mean ± SE) of *P. oceanica* leaves from acidified and control sites pH at Castello Aragonese (CA) and Chiane del Lume (CdL). ANOVA results testing the differences between sites and station are given.

Variable	Vent pH (mean ± SE)		Ambient pH (mean ± SE)		ANOVA	Sum of Squares	*F*-value	*p*-value
	CA (*n*=4)	CdL (*n*=3)	CA (*n*=4)	CdL (*n*=4)				
Shoot density	504.13	307.15	315.12	118.13	Site (df=1)	132705	4.87	<0.05
(m^{-2})	± 117.07	± 57.50	± 58.21	± 56.26	Station (df=1)	144120	5.29	<0.05
Leaf length	9.35	19.08	13.90	23.63	Site (df=1)	25454	262.08	<0.001
(cm)	± 0.21	± 0.47	± 0.58	± 1.06	Station (df=1)	5510	56.74	<0.001
Leaf width	0.912	0.904	1.026	0.952	Site (df=1)	1.86	379.73	<0.001
(cm)	± 0.004	± 0.004	± 0.005	± 0.004	Station (df=1)	0.36	73.80	<0.001
Leaf area index	3.17	5.25	3.01	5.09	Site (df=1)	0.10	0.16	<0.001
(m^2 m^{-2})	± 0.43	± 0.75	± 0.31	± 0.10	Station (df=1)	16.01	27.83	ns

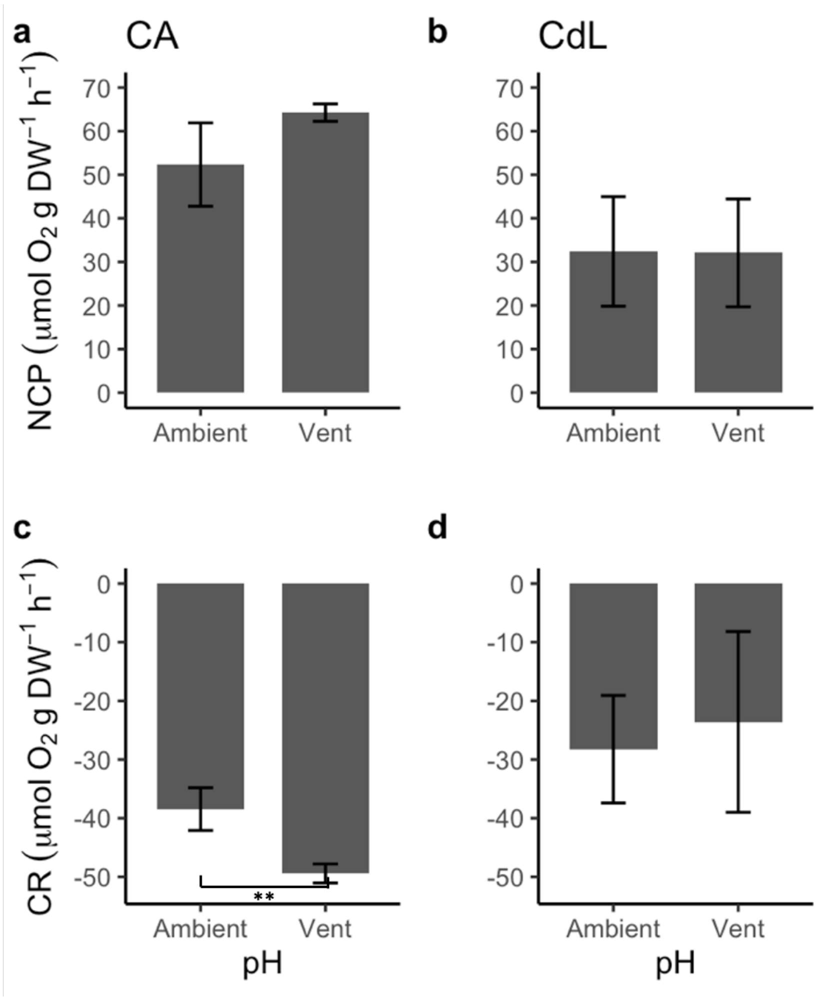

Figure 1. In-situ net community production at CA (a) and CdL (b), and community respiration at CA (c) and CdL (d) at vent and ambient sites, normalized by seagrass leaf biomass (dry weight). Negative values represent oxygen consumption, while positive values show oxygen production. Error bars indicate 95% confidence intervals. Stars show significant differences; number of stars show significance level (* p<0.05, ** p<0.01, *** p<0.001).
